# Retinal tissue develops an inflammatory reaction to tobacco smoke and electronic cigarette vapor in mice

**DOI:** 10.1007/s00109-021-02108-9

**Published:** 2021-07-15

**Authors:** Feng Wang, Stefan Hadzic, Elsa T. Roxlau, Baerbel Fuehler, Annabella Janise-Libawski, Tobias Wimmer, Bo Lei, Shao-Wei Li, Norbert Weissmann, Knut Stieger

**Affiliations:** 1 Department of Ophthalmology, Justus-Liebig-University Giessen, Giessen, Germany; 2 Department of Ophthalmology, Aier School of Ophthalmology, Central South University, Changsha, China; 3 Excellence Cluster Cardiopulmonary Institute (CPI), Member of the German Center for Lung Research (DZL), Universities Giessen and Marburg Lung Center (UGMLC), Justus Liebig University, Giessen, Germany; 4 Henan Eye Institute, Henan Eye Hospital, People′s Hospital of Zhengzhou University, Henan Provincial People′s Hospital, Zhengzhou, China; 5 Beijing Aier-Intech Eye Hospital, Beijing, China

**Keywords:** AMD, C-cigarette smoke, E-cigarette vapor, Inflammatory reaction, Angiogenesis

## Abstract

**Abstract:**

Cigarette smoke has been identified as a major risk factor for the development of age-related macular degeneration (AMD). As an alternative to conventional cigarettes (C-cigarette), electronic cigarettes (E-cigarette) have been globally promoted and are currently widely used. The increasing usage of E-cigarettes raises concerns with regard to short- (2 weeks), medium- (3 months), and long- (8 months) term consequences related to retinal tissue. In this report, a controlled study in mouse models was conducted to probe the comprehensive effects of E-cigarette vapor on retina, retinal pigmented epithelium (RPE), and choroidal tissues by (1) comparing the effects of C-cigarette smoke and E-cigarette vapor on retina separately and (2) determining the effects of E-cigarette vapor on the RPE and analyzing the changes with regard to inflammatory (IL-1β, TNFα, iNOS) and angiogenic (VEGF, PEDF) mediators in retina/RPE/choroid by ELISA assays. The data showed that C-cigarette smoke exposure promoted an inflammatory reaction in the retina in vivo. Mice exposed to E-cigarette (nicotine-free) vapor developed inflammatory and angiogenic reactions more pronounced in RPE and choroid as compared to retinal tissue, while nicotine-containing E-cigarette vapor caused even a more serious reaction. Both inflammatory and pro-angiogenic reactions increased with the extension of exposure time. These results demonstrate that exposure to C-cigarette smoke is harmful to the retina. Likewise, the exposure to E-cigarette vapor (with or without nicotine) increases the occurrence and progression of inflammatory and angiogenic stimuli in the retina, which might also be related to the onset of wet AMD in humans.

**Key messages:**

- C-cigarette smoke exposure promotes an inflammatory reaction in the retina in vivo.
- Mice exposed to E-cigarette (nicotine-free) vapor develop inflammatory and angiogenic reactions more pronounced in RPE and choroid compared to retinal tissue, while nicotine-containing E-cigarette vapor causes even a more serious reaction.
- Both inflammatory and pro-angiogenic reactions increase with the extension of E-cigarette vapor exposure time.

## Introduction

Age-related macular degeneration (AMD) is one of the leading causes of severe vision impairment among the global population [1]. According to the pathological characteristics, AMD is divided into dry and wet based on the absence or presence of choroidal neovascularization (CNV), respectively [2]. Inflammation and the immune dysregulation play crucial roles in the pathogenesis of AMD.

Previous studies have demonstrated that pro-inflammatory cytokines interleukin 1 beta (IL-1β) and tumor necrosis factor alpha (TNF-α) can promote angiogenesis in choroidal neovascular membranes [3, 4], and are able to disrupt the structure and function of the outer and inner blood-retinal barrier (BRB) [5–7], leading to the progression of wet AMD. Moreover, the inflammatory enzyme inducible nitric oxide synthase (iNOS), which can produce large amounts of nitric oxide (NO) enduringly, is upregulated in pathological conditions such as inflammation or in the presence of certain cytokines (e.g., TNF, interleukins). Excessive NO not only causes oxidative stress, but also reacts with superoxide anion radicals forming peroxynitrite, which further contributes to vascular damage and promotes the development of AMD or other retinopathies [8].

The pro-angiogenic cytokine vascular endothelial growth factor (VEGF) participates in the complex regulation of angiogenesis and vascular permeability, and is the crucial promoter for CNV. However, an impaired VEGF signaling results in a dysfunctional retinal pigmented epithelium (RPE)/Bruch’s membrane (BrMb), which is presumably involved in the pathogenesis of dry AMD [9]. In contrast, pigment epithelium-derived factor (PEDF) is an anti-angiogenic and neuroprotective factor. It affects the proliferation and the oxidative stress state of choroidal endothelial cells [10]. A balance between VEGF and PEDF has been demonstrated in the RPE and choroid, and a disruption of this balance would result in pathological angiogenesis [11, 12].

Among numerous risk factors of AMD, cigarette smoke is the largest single preventable factor [13]. The epidemiological evidences highly support the causal association between cigarette smoke and progression of AMD [14]. E-cigarettes are battery-powered devices delivering vapor to the user by heating e-liquid that normally contains solvents, flavoring agents, with or without nicotine [15]. As advertised being the “less dangerous” alternative, E-cigarettes are supposed to generate less toxicants. However, the effect of E-cigarette vapor on the retinal tissue has so far not been analyzed.

Although some aerosol studies revealed that the particles generated from E-cigarette have a significantly lower biological activity than C-cigarette smoke [16, 17], the increasing evidences suggest that these ultrafine particles still can induce inflammatory reactions, and the usage of E-cigarettes would generate toxicity and induce the release of inflammatory mediators [18–20]. Animal studies also showed that nicotine-containing E-cigarette vapor exposure could increase the pulmonary inflammation and oxidative stress in mice [21, 22]. To date, the scientific evidence regarding the health effects of E-cigarette vapor on the retina or fundus is still limited, thus posing the question of whether E-cigarette vapor is similarly dangerous as compared to C-cigarette smoke.

Herein, we assess the effect of C-cigarette smoke and E-cigarette vapor on the retina by molecular investigation in the smoker mouse model. Furthermore, the effect of E-cigarette vapor on RPE and choroid was determined and the changes with regard to inflammatory and angiogenic mediators in retina/RPE/choroid were analyzed in order to evaluate a potential relationship between E-cigarette vapor exposure and the induction of inflammatory angiogenic effects in mice, which might be related to the onset of wet AMD in humans.

## Materials and methods

**Fig. 1:**
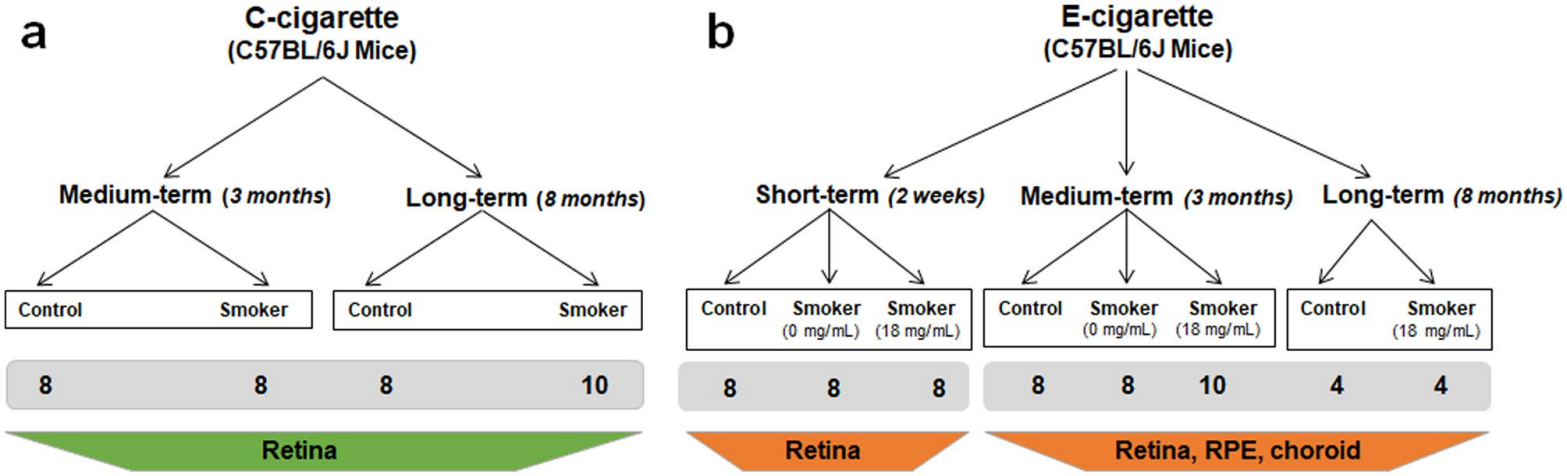
Overview of the experimental groups. **a** C-cigarette exposure groups contain medium- and long-term subgroups and the corresponding control mice. **b** E-cigarette exposure groups contain short-, medium-, and long-term subgroups, wherein nicotine-free E-cigarette and nicotine-containing E-cigarette were used with the exception of the long-term subgroup, in which only nicotine-containing E-cigarettes was used. The number of eyes per group is listed in the grey box below each group

The data presented in this paper are part of a large multidisciplinary experimental setup targeted at describing the effect of smoke and vapor exposure on different tissues of the body, in particular the lung. Mice used in the experiments were C57BL/6J from our own colony or directly purchased from Charles River Deutschland, Sulzfeld, Germany. Animals were housed under a 12:12 h, light-dark cycle and food and water supply ad libitum during experiment. Overview of the experimental groups is displayed in Fig. 1.

### Experimental animals and smoke exposure

Retina tissue was harvested in the C-cigarette exposure groups and E-cigarette short exposure subgroup. In E-cigarette medium and long-term exposure subgroups, all the retina, RPE, and choroid were harvested. In each group, retinas were pooled in order to perform the different assays presented in this paper. In the E-cigarette setup, mice were exposed to the vapor of nicotine-free liquid or liquid containing 18 mg/ml nicotine. The only exception is the long-term exposure time-point, where mice were exposed only to vapor from nicotine containing liquid. Age-matched mice that were kept under identical conditions without cigarette-smoke or -vapor exposure were used as corresponding controls.

### C-cigarette smoke exposure

Male C57BL/6J mice (14 ± 2 weeks old) were divided into two subgroups with different exposure time (Fig. 1a). Whole body exposure to the mainstream smoke of 3R4F cigarettes (Kentucky Tobacco Research and Development Centre, USA, containing 0.7 mg nicotine per cigarette) generated by a smoke generator (Burkhart, Wedel, Germany) was done as previously described [23–25]. The particle concentration was adjusted to 200 mg/m^3^, and mice were exposed for 6 h per day, 5 days per week for a period of 3 (medium-term), or 8 (long-term) months [26]. Mice in the control group remained in the room air environment, while the other conditions were similar to the corresponding experimental group.

### E-cigarette vapor exposure

Male C57BL/6J mice (14 ± 2 weeks old) were exposed in a whole-body manner to E-cigarette vapor for 6 h per day, 5 days per week, using inExpose inhalation exposure system (SCIREQ, Montreal, Canada). E-cigarette vapor is generated by Joyetech eVic-VTC Mini E-cigarettes (Joyetech) that are integrated into the inExpose inhalation exposure system (SCIREQ Scientific Respiratory Equipment Inc.). Commercially available E-cigarette basic liquid (Avoria GmbH, Nuremberg, Germany) containing 55% propylene glycol, 35% glycerol, and 10% water was used. E-cigarette liquid is vaporized at 2 puffs per minute (2 s evaporation per puff) and hence 720 puffs in total for one day (6 h). There are three different subgroups in the E-cigarette vaping setup (Fig. 1b) with different exposure time of 2 weeks, 3 months, and 8 months as short-, medium-, and long-term subgroups, respectively. In order to evaluate the effect of nicotine on retina/RPE/choroid, both the short- and medium-term subgroups were further divided into two independent vaping subgroups using different kinds of E-cigarette liquids (nicotine-free and containing 18 mg/ml nicotine) without flavoring reagents.

### Retina, RPE, and choroid/scleral dissection

Following euthanasia, the eyes were enucleated and placed in 1× PBS buffer on ice immediately. The retina was removed carefully by cutting the optic nerve. The cup-shaped half eyeball (composed of the RPE, choroidal, and scleral tissues was cut into four small flaps from the edge to the direction of the optic nerve vertically. The remaining tissue was flattened, and transferred into a 1.5-mL centrifuge tube which contained ice bath-cooled 100 µL of RIPA protein lysis buffer with protease inhibitor. After complete immersion in the lysis buffer, the centrifuge tubes were placed on ice for incubation for 1 h. The centrifuge tubes were tapped several times to help RPE layer to be separated from choroid/sclera. In the later stage of incubation, a brown clump (i.e., RPE layer) was observed as detached from the choroid/sclera, which was removed into a separate tube [27]. All samples were immediately stored at −80 °C until further use. All retinas, RPE and choroid were pooled per subgroup in order to obtain sufficient material to perform all assays (see below).

### Protein extraction and enzyme-linked immunosorbent assay

Proteins were isolated using AllPrep RNA/Protein Kit (80404, QIAGEN, Germany) and used to quantify the levels of cytokine IL-1β and VEGF, which were analyzed by mouse IL-1beta/IL-1F2 DuoSet and mouse VEGF DuoSet ELISA kit (R&D Systems), and levels of PEDF, iNOS, and TNF-α proteins by using Mouse Pigment epithelium-derived factor (PEDF) ELISA Kit (KTE70449, Abbkine, China), Inducible Nitric Oxide Synthase (iNOS), ELISA Kit (MBS030771, My BioSource, USA), and mouse TNF-α DuoSet ELISA kit (R&D Systems), respectively. All assays were performed according to related manufacturer’s protocol. All samples were assayed in duplicate. The medium- and long-term control mice from C-cigarette and E-cigarette exposure groups were approximately at the same age, same genetic background and kept in similar conditions, thus the control data were merged.with sufficient accuracy

In case of very low protein level detection, i.e., when measuring IL-1β and VEGF, we have extended the standard curve by further diluting the lowest standard sample and were able to calculate the lower limit of detection based on DIN 32645 as being 1.95 pg/ml with sufficient accuracy (R^2^ ≥ 0.8) in both cases.

**Table 1:** Detection range of each protein by ELISA

Protein name	Standard curve range
IL-1β^a^	1.95−1000 pg/mL
iNOS^b^	0.1−8 U/L (sensitivity 0.01U/L)
TNF-α^b^	31.3−2000 pg/mL
VEGF^a^	1.95−1000 pg/mL
PEDF^b^	10−320 μg/L (the minimum detectable dose is typically less than 1 μg/L)

^a^Own validation

^b^As published by the manufacturer

### Data statistics

Values were expressed as mean ± SD (standard deviation). One or two-way analysis of variance (1 or 2 way-ANOVA) or multiple t tests were used to determine statistical significance. All statistics were carried out using GraphPad Prism 7 (GraphPad Software Inc, SanDiego, CA, USA), *P* < 0.05 was defined as statistically significant (Table 1).

## Results

### The medium- and long-term effects of C-cigarette smoke exposure on the retina

**Fig. 2:**
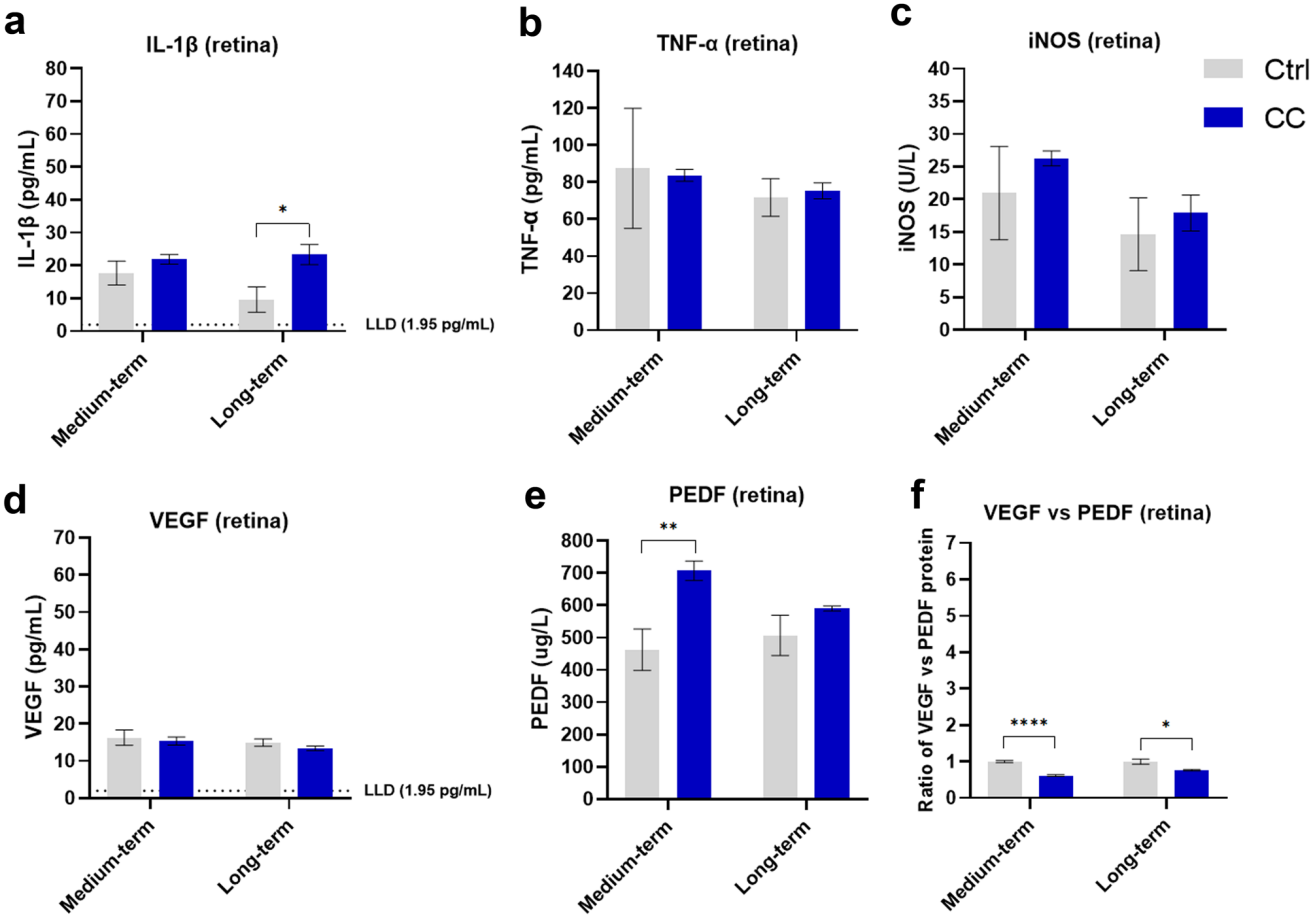
Protein levels in the retina of mice from the C-cigarette smoke exposure (medium-, long-term) subgroups. Data presented are from factors IL-1β (**a**), iNOS (**b**), TNF-α (**c**) and VEGF (**d**), and PEDF (**e**). Ratio of VEGF vs PEDF reflects the changes to the equilibrium of both factors at the RPE/retina interface (**f**) reflects the comprehensive effect of pro-angiogenic. Data are presented as mean ± SD. *P < 0.05, **P < 0.01, ***P < 0.001, ****P < 0.0001. LLD lower limit of detection

In retinas from medium-term C-cigarette smoke exposure subgroup, the level of PEDF (Fig. 2e) slightly increased, and the ratio of VEGF *vs.* PEDF (Fig. 2f) decreased by about 40% compared to control mice, but there is no significant difference for the inflammation-related cytokine protein level between smoker and control mice. In retinas from long-term C-cigarette smoke exposure subgroup, the level of IL-1β (Fig. 2a) was significantly higher than that in control mice (about 2.4 folds), while the ratio of VEGF *vs.* PEDF (Fig. 2f) slightly decreased.

### The short-, medium-, and long-term effects of E-cigarette vapor (with or without nicotine) exposure on the retina

**Fig. 3:**
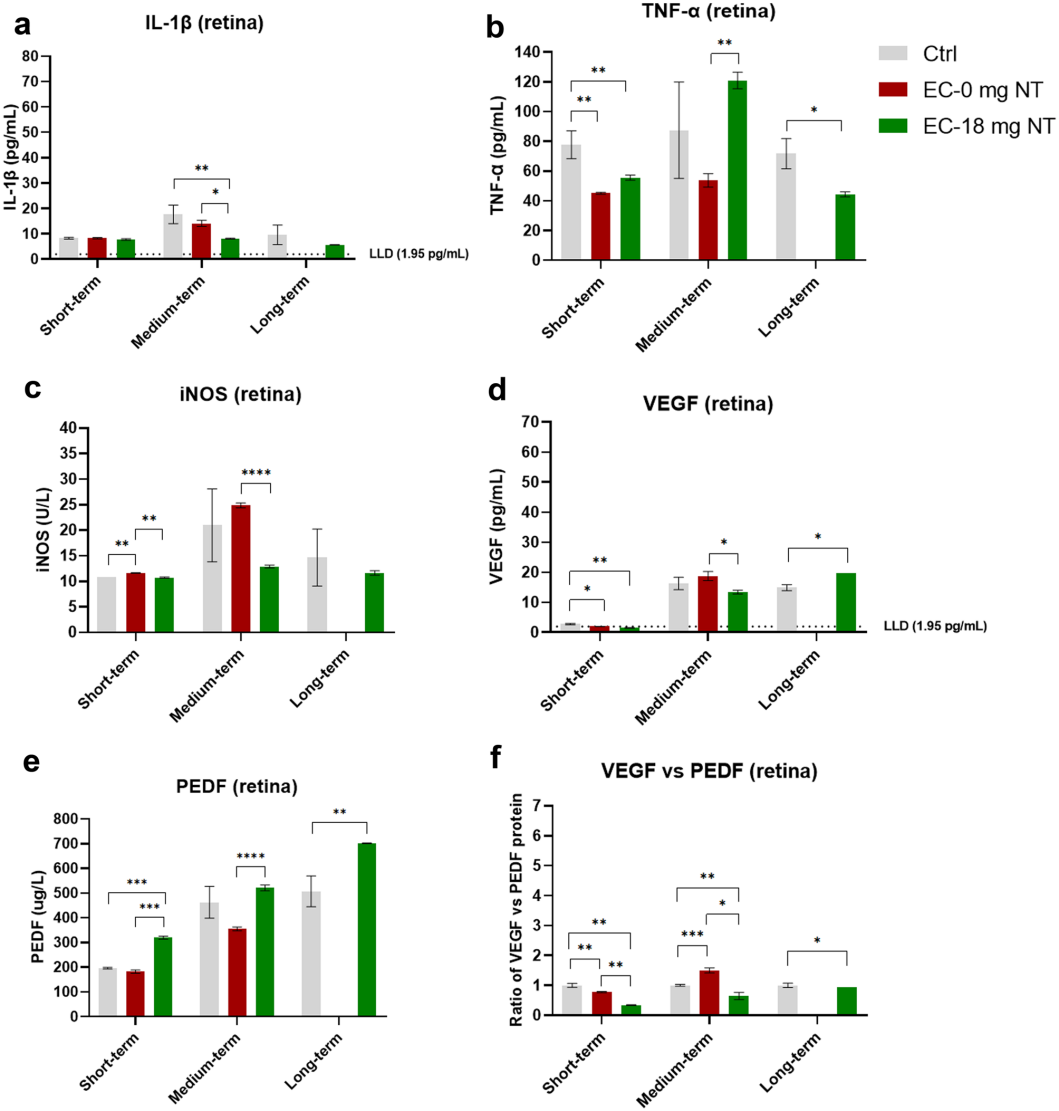
Protein levels in the retina of mice from the E-cigarette vapor exposure (short-, medium-, long-term) subgroups. Data presented are from factors IL-1β (**a**), iNOS (**b**), TNF-α (**c**) and VEGF (**d**), and PEDF (**e**). Ratio of VEGF vs PEDF reflects the changes to the equilibrium of both factors at the RPE/retina interface (**f**) reflects the comprehensive effect of pro-angiogenic. Data are presented as mean ± SD. *P < 0.05, **P < 0.01, ***P < 0.001, ****P < 0.0001. LLD lower limit of detection

In the short-term E-cigarette vapor exposure subgroup, the expression of iNOS (Fig. 3c) in the retina in nicotine-free subgroup was increased, while the level of TNF-α (Fig. 3b) and the ratio of VEGF *vs.* PEDF (Fig. 3f) decreased significantly. Results from the nicotine-containing E-cigarette subgroup showed a decreased level of TNF-α (Fig. 3b) and a down-regulated ratio of VEGF *vs.* PEDF (Fig. 3f) in the retina from vapor exposed mice comparing with control mice.

In the medium-term E-cigarette vapor exposure subgroups, the ratio of VEGF *vs.* PEDF (Fig. 3f) was significantly increased in nicotine-free E-cigarette subgroup. In contrast, the results from the nicotine-containing subgroup indicated that the expression of IL-1β (Fig. 3a) and the ratio of VEGF *vs.* PEDF (Fig. 3f) significantly decreased.

Interestingly, long-term nicotine-containing E-cigarette vapor exposure reduced the expression of TNF-α (Fig. 3b), and down-regulated the ratio of VEGF *vs.* PEDF as well (Fig. 3f).

### The medium- and long-term effects of E-cigarette vapor (with or without nicotine) exposure on the RPE and choroid

**Fig. 4:**
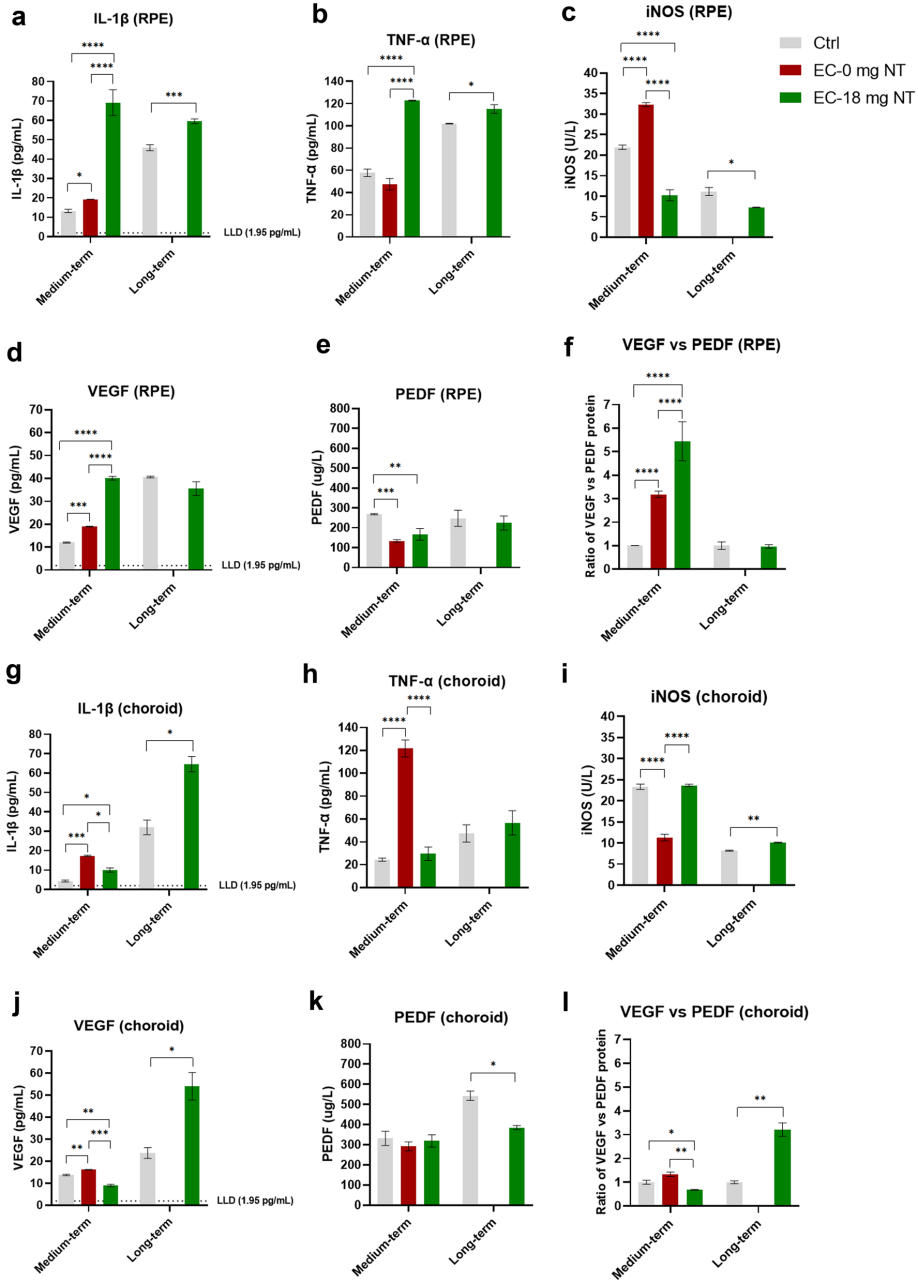
Protein levels in the RPE (**a**−**f**) and choroid (**g**−**l**) of mice from the E-cigarette exposure (medium-, long-term) subgroups. Data presented are from factors IL-1β (**a**, **g**), iNOS (**b**, **h**), TNF-α (**c**, **i**), VEGF (**d**, **j**), and PEDF (**e**, **k**). Ratio of VEGF vs PEDF reflects the changes to the equilibrium of both factors at the RPE/retina interface (**f, l**) reflects the comprehensive effect of pro-angiogenic. All the data are presented as mean ± SD. *P < 0.05, **P < 0.01, ***P < 0.001, ****P < 0.0001. LLD, lower limit of detection

Surprisingly, in the RPE, after medium-term nicotine-free E-cigarette vapor exposure, the protein levels of IL-1β (Fig. 4a), iNOS (Fig. 4c), and VEGF (Fig. 4d) were up-regulated, and the ratio of VEGF *vs.* PEDF (Fig. 4f) increased significantly as well. Simultaneously, the results from the nicotine-containing subgroup indicated that both levels of IL-1β (Fig. 4a) and TNF-α (Fig. 4b) increased significantly. Besides, the increase of the ratio of VEGF *vs.* PEDF (Fig. 4f) was more evident comparing with the nicotine-free subgroup. In the long-term exposure subgroup, the changes of protein levels of pro-inflammation mediators were similar but less significant than that from medium-term exposure subgroup. However, there was no significant change for the ratio of VEGF *vs.* PEDF (Fig. 4f).

In the choroid, after medium-term nicotine-free E-cigarette vapor exposure, the levels of IL-1β (Fig. 4g), TNF-α (Fig. 4h), and VEGF (Fig. 4j) all increased significantly. The results from the nicotine-containing subgroup showed a slight increase of IL-1β (Fig. 4g), and a significantly decreased ratio of VEGF *vs.* PEDF (Fig. 4l). In addition, long-term exposure of nicotine-containing E-cigarette vapor resulted in higher levels of IL-1β (Fig. 4g), iNOS (Fig. 4i), and an increased ratio of VEGF *vs.* PEDF (Fig. 4l).

### Comparison of the effect of C-cigarette smoke with E-cigarette vapor (nicotine-free or nicotine-containing) on retinal tissue

**Fig. 5:**
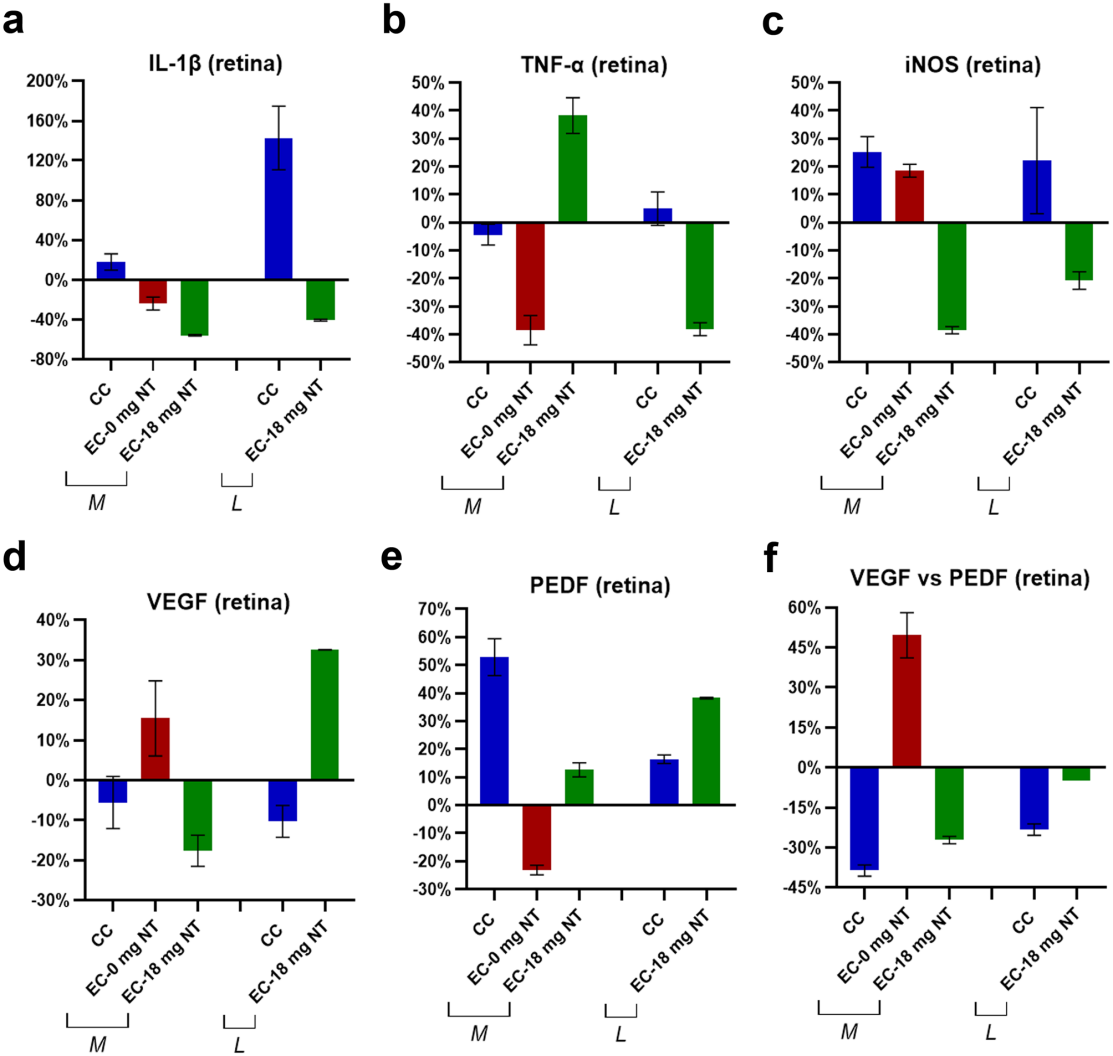
Comparison of the percentage changes in inflammation-related (**a** IL-1β, **b** TNF-α, **c** iNOS) and angiogenesis-related cytokines (**d** VEGF, **e** PEDF, **f** ratio of VEGF *vs.* PEDF). Protein levels were compared between C-cigarette smoke and E-cigarette vapor subgroups. CC: C-cigarette smoke, EC-0 mg NT: E-cigarette (nicotine free) vapor, EC-18 mg NT: E-cigarette (nicotine containing) vapor, M: medium-term, L: long-term

In medium-term exposure groups, the expression of the anti-angiogenic mediator PEDF (Fig. 5e) in retinal tissues exposed to C-cigarette smoke was increased by 53%, along with a decreased ratio of VEGF *vs.* PEDF (Fig. 5f) by 39%. Surprisingly, PEDF (Fig. 5e) in the nicotine free E-cigarette subgroup changed in an opposite trend, leading to an increased ratio of VEGF *vs.* PEDF (Fig. 5f) by 50% rather than decreased. In the nicotine-containing E-cigarette subgroup, the expression level of IL-1β (Fig. 5a) decreased by 56% after exposure, while TNF-α (Fig. 5b) increased by 38%. Besides that, the trends in the regulation of angiogenic-related factors were similar to the C-cigarette subgroup.

In long-term exposure subgroups, the trends in changes in the C-cigarette exposure subgroup were similar to the medium-term group. The most significant change was the upregulated IL-1β (Fig. 5a) protein by 143%, while the change in the ratio of VEGF *vs.* PEDF (Fig. 5f) was smaller. In nicotine-containing E-cigarette group, inflammatory-related cytokines IL-1β (Fig. 5a), TNF-α (Fig. 5b), and iNOS (Fig. 5c) were all slightly down-regulated. In addition, the expression levels of VEGF (Fig. 5d) and PEDF (Fig. 5e) were increased simultaneously, giving a decreased ratio of VEGF *vs.* PEDF (Fig. 5f) by 4.8%.

## Discussion

Most of the reported studies about the association of smoking with AMD are mainly focused on the effect of smoking on RPE and scarcely on the retina. In the present study, the effect of smoking on the retina, RPE and choroidal tissue was investigated by studying the protein levels of different cytokines after medium- and long-term exposure to C-cigarette smoke, as well as short-, medium-, and long-term exposure to E-cigarette vapor (with or without nicotine). With regard to E-cigarette vapor, the medium- and long-term exposure experiments were conducted to simulate the human’s vaping habits and to investigate the association of E-cigarette vapor with increased inflammation and angiogenesis. The short-term exposure was performed as well to assess the immediate adverse effects of E-cigarette vapor on retina.

The pathogeneses of the two types of AMD are not alike. Dry AMD is assumed to begin with RPE dysfunction followed by the dysfunction/loss of photoreceptors and choriocapillaris. In wet AMD, it probably starts with the dysfunction/loss of choroidal vasculature alone or with RPE layer together, followed by the accumulation of pro-inflammatory mediators in choriocapillaris, and the subsequent production of excessive angiogenic substances by RPE because of hypoxia, which will result in angiogenesis from the choroidal vessels into the retina (CNV), and photoreceptor loss [28, 29]. In the present study, the comprehensive results from retina, RPE and choroid after medium-term exposure to E-cigarette vapor revealed that both nicotine-containing and nicotine-free E-cigarette vapor could stimulate the expression of pro-inflammatory and angiogenic mediators and accumulate in the RPE and choroidal tissues. This is likely the cause that leads to an inflammatory response in these tissues and induces CNV. Interestingly, such results are in line with the pathogenesis of wet AMD as mentioned above, which suggests that even without nicotine or flavoring agents, E-cigarette vapor only derived from basic solvents (propylene glycol, vegetable glycerin) still can promote the occurrence and progression of wet AMD. In addition, the adverse effects, which are stimulated by nicotine-containing E-cigarette vapor on the RPE, are stronger than that by nicotine-free one. As the only difference between these two E-cigarette vapors is the nicotine component, it further corroborates the notion that the nicotine component enhances the harmful effect of basic solvents on RPE and choroid. As shown in Fig. 4f, the relative change of the ratio of VEGF *vs.* PEDF in the nicotine-containing subgroup is about 1.7 times higher than the value in the nicotine-free subgroup. This result is consistent with the experimental study by Pons and colleagues, wherein they confirmed that nicotine could increase the ratio of VEGF *vs.* PEDF by combining with nicotinic acetylcholine receptors in RPE, which is critical in the development of wet AMD for second-hand smokers [30]. Previous studies have demonstrated that nicotine is an agent with pro-angiogenic effect and can stimulate the proliferation of CNV [31], and the impact of nicotine on the expression of pro-angiogenic and inflammatory mediators has also been reported [32]. The study by Bekki and colleagues showed that heating the basic solvents could generate some carbonyl compounds such as formaldehyde, acetaldehyde, acetone, and acrolein [33]. Other studies suggested that acrolein also exists in cigarette smoke, which could potentially induce inflammatory reaction from macrophages and epithelial cells [34, 35]. All these reported findings could rationalize our results from nicotine-containing E-cigarette vapor exposure experiments, that even the vapor derived from e-liquid only containing the basic solvents can stimulate the expression of pro-inflammatory and angiogenic mediators in RPE, as well as pro-inflammatory mediators in choroid. Based on these reports, we speculate that the acrolein or other relative carbonyl compounds in the vapor from solvents induced the expression of pro-inflammatory mediators from RPE cells and the choroidal capillary endothelial cells or some unique inflammatory cells. Nevertheless, further longitudinal studies are needed to unveil how the nicotine-free E-cigarette vapor causes angiogenesis.

By comparing the data from medium-term to long-term E-cigarette vapor exposure, we observed that the predominantly impaired tissues changed from RPE and choroid to choroid alone. Moreover, the levels of VEGF and PEDF decreased dramatically in RPE, and turned even lower than in related control mice. According to the pathological progression of wet AMD, it is assumed that with the extension of exposure period, the damage of RPE might become more serious, and the RPE layer becomes dysfunctional or even apoptotic/necrotic, leading to a decreased ability to produce pro-inflammatory and angiogenic factors. On the other hand, due to the increasing loss of choroidal blood vessels, the hypoxia of choroidal capillaries becomes more serious [36], which would stimulate the choroidal capillary endothelial cells to produce more angiogenic substances like VEGF, and hence, the expression of antiangiogenic mediator PEDF decreases, promoting the generation of CNV in lesions at the RPE/retina interface, which is a hallmark sign in the development of wet AMD in humans.

In addition, our results indicate that after short-term exposure to E-cigarette vapor (both nicotine-free and nicotine-containing), in retinal tissue, the anti-angiogenic pathway is activated and no significant change occurred with regard to the expression of pro-inflammatory mediators. This suggests that short-term exposure to E-cigarette vapor only has limited pro-inflammatory effect on retinal tissue. Furthermore, by summarizing the medium- and long-term observation, it is assumed that both E-cigarette vapors with and without nicotine have limited damage on retina, but more so on RPE and choroid.

To date, the exact mechanism of cigarette smoking on retina/RPE/choroid is still unknown. Nevertheless, some reviews have summarized that cigarette smoking can increase the oxidative stress burden and hence induce the inflammation response on RPE and choroid, causing the damage to retina/RPE. Furthermore, impairing the choroidal blood flow and decreasing the perfusion pressure could result in hypoxia and promote angiogenesis, and eventually cause the development of AMD [37–39]. Another review elaborated that E-cigarette vapor exposure not only disrupt pulmonary homeostasis but also can increase inflammatory response and oxidative stress via airway [40]. Although there are no specialized studies regarding the mechanism of E-cigarette vapor on the retina, based on the respiratory system studies, we assumed that the vapor could also cause an inflammatory response and oxidative stress to the fundus similar to C-cigarette smoke.

With regard to limitations of our study, it should be considered that whole-body exposure was performed in this mouse model. The anterior tissue of the ocular surface such as cornea as well as skin surrounding the eye has been exposed to the smoke or vapor, which might have an impact on our results. However, anterior/posterior diffusion of molecules in the eye is not easily possible due to the presence of physiological barriers. Therefore, since this exposure is also similar to human smoke/vapor exposure, it should not interfere significantly with the data presented here. A second limitation is the relatively low number of eyes analyzed in some of the subgroups (E-cigarette, long-term exposure). This renders the interpretation of the respective data less robust. However, these initial data on a small number of animals provide a first view on these yet under-investigated mechanisms and pave the way for further experiments with an increased number of animals. A third limitation is the fact that values for VEGF and IL-1ß were sometimes very low and below the detection range as published by the manufacturers. In order to generate valuable data also in these samples, we have extended the standard curve of the ELISA and calculated the lower limit of detection separately. This way, we have obtained values that have to be considered with more caution compared to those obtained within the standard curve offered by the manufacturer.

In conclusion, by providing molecular experimental evidences, our study demonstrated for the first time that exposure to E-cigarette vapor (with or without nicotine) induces the occurrence and progression of inflammatory and angiogenic effects in the retina. The nicotine component in vapor likely enhances the harmful effect of the basic humectants. In addition, with the extension of exposure period, nicotine-containing E-cigarette vapor further increases the likelihood of the generation of pathologic effects similar to those of wet AMD.

## Data Availability

The data sets generated and/or analyzed during the current study are available from the corresponding author on reasonable request.
